# Association Mapping and Transcriptome Analysis Reveal the Genetic Architecture of Maize Kernel Size

**DOI:** 10.3389/fpls.2021.632788

**Published:** 2021-03-18

**Authors:** Juan Ma, Lifeng Wang, Yanyong Cao, Hao Wang, Huiyong Li

**Affiliations:** Institute of Cereal Crops, Henan Academy of Agricultural Sciences, Zhengzhou, China

**Keywords:** kernel size, kernel development, genome-wide association study, transcriptome, differentially expressed genes

## Abstract

Kernel length, kernel width, and kernel thickness are important traits affecting grain yield and product quality. Here, the genetic architecture of the three kernel size traits was dissected in an association panel of 309 maize inbred lines using four statistical methods. Forty-two significant single nucleotide polymorphisms (SNPs; *p* < 1.72E-05) and 70 genes for the three traits were identified under five environments. One and eight SNPs were co-detected in two environments and by at least two methods, respectively, and they explained 5.87–9.59% of the phenotypic variation. Comparing the transcriptomes of two inbred lines with contrasting seed size, three and eight genes identified in the association panel showed significantly differential expression between the two genotypes at 15 and 39 days after pollination, respectively. Ten and 17 genes identified by a genome-wide association study were significantly differentially expressed between the two development stages in the two genotypes. Combining environment−/method-stable SNPs and differential expression analysis, ribosomal protein L7, jasmonate-regulated gene 21, serine/threonine-protein kinase RUNKEL, AP2-EREBP-transcription factor 16, and Zm00001d035222 (cell wall protein IFF6-like) were important candidate genes for maize kernel size and development.

## Introduction

Maize is one of the most important crops and is widely used as staple food, animal feed, and raw materials. Grain yield improvement is a longstanding breeding goal in maize. Kernel size traits, including kernel length (KL), kernel width (KW), and kernel thickness (KT), largely affect yield component kernel weight and product quality. In addition, large kernels have a favorable seed vigor and, finally, promote yield. Therefore, it is important to elucidate the genetic architecture of kernel size.

Using linkage mapping, many quantitative trait loci (QTLs) for kernel size traits have been identified in F_2:3_ families (Liu et al., 2014; Li et al., 2019), doubled haploid (DH; Shi et al., 2017; Liu et al., 2020a), recombinant inbred line (RIL; Raihan et al., 2016; Liu et al., 2017; Zhang et al., 2017), three-way cross (Jiang et al., 2015), four-way cross (Chen et al., 2016), and immortalized F_2_ population (Liu et al., 2020b) with different backgrounds. For instance, Liu et al. (2014) identified 6, 16, and 18 QTLs for KL, KW, and KT, respectively, accounting for 0.84–20.51% of phenotypic variation in an F_2:3_ population. In intermated B73 × Mo17 Syn10 DH population, Liu et al. (2020a) found 50 QTLs for KL, KW, and KT, of which 18 QTLs were detected in at least two environments. Liu et al. (2017) identified 729 QTLs for KL, KW, KT, and kernel weight in 10 RIL populations using three statistical models.

Genome-wide association study (GWAS) is an effective approach for analyzing the genetic basis of complex traits. GWAS results are easily influenced by population structure and rare variants in natural populations. Therefore, many statistical methods have been developed to improve the power for identifying phenotype-genotype associations such as single-locus mixed linear model (MLM; Yu et al., 2006), compressed MLM (CMLM; Zhang et al., 2010), and settlement of MLM under progressively exclusive relationship (SUPER; Wang et al., 2014), and multi-locus methods mrMLM (Wang et al., 2016), multiple locus mixed linear model (MLMM; Segura et al., 2012), and fixed and random model circulating probability unification (FarmCPU; Liu et al., 2016). The MLM method has proven useful in controlling for population structure and relatedness within GWAS (Yu et al., 2006). To improve the statistical power and solve the confounding problem for MLM methods, the CMLM method decreases the effective sample size by clustering individuals into groups that is fitted as random effects (Zhang et al., 2010). SUPER remarkably increases the statistical power and solves the computational problem using influential bins which are represented by the most significant markers (Wang et al., 2014). MLMM utilizes multiple markers simultaneously as covariates in a stepwise MLM to partially eliminate the confounding between kinship and testing markers (Segura et al., 2012; Liu et al., 2016). FarmCPU divides MLMM into two parts, i.e., fixed effect model and random effect model, and uses them iteratively (Liu et al., 2016). Many studies have identified variations of single nucleotide polymorphisms (SNPs) for kernel size traits in different association panels using these methods (Zhang et al., 2017; Li et al., 2019; Liu et al., 2020a) in maize. Li et al. (2019) identified 18, 19, and 7 significant SNPs for KL, KW, and KT in 639 inbred lines selected from a nested association mapping population using FarmCPU. Twenty-one SNPs were detected for the three traits, of which two SNPs were detected by CMLM, one by MLM, and 20 by FarmCPU (Liu et al., 2020a). In addition, Zhang et al. (2017) found that a stable locus PKS2 affecting kernel shape was detected on chromosome 2 by combined linkage and association mapping.

A large number of QTLs or SNPs were detected using the two mapping approaches, and only a few genes influencing kernel size traits have been cloned. For instance, *ZmCKX10*, encoding cytokinin oxidase, was cloned by fine mapping of a major QTL (qKL1.07) for KL (Qin et al., 2016). Although numerous functional genes have been reported to regulate kernel development through mutant analysis (Fu et al., 2002; Kang et al., 2009; Li et al., 2014; Yang et al., 2017; Zhu et al., 2019; Dai et al., 2020), the application of these functional genes is limited for lacking superior allelic variations when using marker-assisted selection breeding (Liu et al., 2020a).

Transcriptome has also been employed to detect the underlying genetic architecture responsible for phenotypic variations. By integrating GWAS, expression quantitative trait loci (eQTL), and quantitative trait transcript analyses, Pang et al. (2019) identified 137 putative KL-related genes at 5 days after pollination (DAP5) and an eQTL that overlapped the locus encoding a maize homolog of m6A methylation reader protein ECT2 of *Arabidopsis*. Transcriptome analysis not only reveals a large number of genes associated with kernel size and development but also some biological processes and signaling pathways including DNA methylation, ovule development, cell cycle, cell division, ubiquitin, phytohormone signaling pathways, and transcriptional regulatory factors during seed, endosperm, and embryo development in maize (Sekhon et al., 2014; Zhang et al., 2016). These studies provide extensive information for genes and regulatory networks, which are helpful to dissecting the genetic architecture of kernel size traits.

To date, the dissection of kernel size traits by integrating GWAS and transcriptome analysis is rare. In the present study, we used an association panel including 309 inbred lines to identify significant SNPs and candidate genes for KL, KW, and KT in multi-environments using four GWAS methods. We also identified differentially expressed genes of two inbred lines with contrasting seed size that were selected from the association panel during two seed development stages. We finally determined consistent genes associated with kernel size by combined GWAS and transcriptome analysis.

## Materials and Methods

### Experimental Design, Phenotyping, and Analysis

A total of 309 maize inbred lines were assembled into a panel. The panel was comprised of 128 China core germplasms, 16 new selected inbred lines, and 165 US public inbred lines whose plant variety protection had expired (provided by the China National Modern Corn Industry Technology System). They were planted in Yuanyang (YY, 35.012 N, 113.704 E), Dancheng (DC, 33.646 N, 115.257 E), and Sanya (SY, 18.381 N, 109.183 E) experimental stations of Henan Academy of Agricultural Sciences in 2017. In 2019, the association panel was planted in YY station. The field experiment was arranged in a randomized complete block design with three replicates. Each inbred line was grown in two rows with 15 plants, 0.60 m in row spacing, and 0.25 m in plant spacing. Best linear unbiased estimate (BLUE) values of each trait in the four environments were calculated by the software QTL IciMapping v4.0 (Meng et al., 2015) and were used as phenotypes of the combined environment. Three well-developed ears were harvested for KL, KW, and KT measurement. An automatic variety test system for maize ear (National Engineering Research Center for Information Technology in Agriculture, Beijing, China) was used to measure the three traits with 50 randomly selected kernels from each line.

Pearson correlation was calculated by R software (R Development Core Team, 2015). For single environment and multi-environments, the broad-sense heritability at per mean level was calculated by QTL IciMapping v4.0. Genotype, environment, block within environment, and genotype and environment interactions (GEI) were used in the multi-environment analysis of variance (ANOVA) model, which was performed by QTL IciMapping v4.0.

### Genotyping, Population Structure, Kinship, and Genome-Wide Association Analysis

The association population was sequenced by genotyping-by-sequencing technology (Novogene Bioinformation Technology Co., Ltd., Beijing, China). The reads were aligned against the maize B73 genome^1^ using the BWA software. SNPs were identified using SAMtools (Li et al., 2009). A total of 58,129 SNPs were used for GWAS after filtering SNPs with minor allele frequency (MAF) < 0.05, missing rate > 0.10, and heterozygous rate > 0.10. The Centered_IBS method in TASSEL v5.2.60 (Bradbury et al., 2007) was used to calculate kinship matrix between lines. The Bayesian Markov Chain Monte Carlo (MCMC) method in Structure v2.3.4 (Pritchard et al., 2000) was used to estimate the subgroups (*K*). *K* was set from 1 to 8 with three-time iterations. Length of burnin period was 5,000 and the number of MCMC replicates after burnin was 50,000. The results were visualized by Structure Harvester (Earl and vonHoldt, 2012), and delta *K* was used to determine the optimal number of subgroups. Two subgroups were revealed in the panel (Supplementary Figure S1). One subgroup with 79 lines included Stiff Stalk, whereas the other was a mixed heterotic group which was mainly comprised of non-Stiff Stalk and Reid. To balance positive and negative significant SNPs, two single-locus MLM models, namely CMLM and SUPER, and two multi-locus methods, namely MLMM and FarmCPU, were conducted in GAPIT package (Lipka et al., 2012). The kinship and the population structure (*Q* matrix) were incorporated in the four methods. All parameters were set by default. Stringent Bonferroni correction is usually adopted for multiple testing correction in a single-locus model, whereas no multiple testing correction is needed in multi-locus methods (Zhang et al., 2019). Therefore, a moderate threshold for significant SNPs was set at 1/total number of SNPs (58,129) = 1.72E-05 for the four methods, which was used by previous studies (Wang et al., 2015; Ma et al., 2016; Zhang et al., 2017; Zhu et al., 2018). Phenotypic variation explained (PVE) of significant SNPs identified from FarmCPU, MLMM, and SUPER was calculated according to a previous study (Liu et al., 2020a), and that of CMLM was given by GAPIT. Candidate genes were identified from 50 kb upstream and downstream of each significant SNP by ANNOVAR (Wang et al., 2010).

### Transcriptome Sequencing and Differentially Expressed Analysis

To validate the candidate loci identified by GWAS, we selected two inbred lines from the association panel mainly according to the KL and KW value in SY2017 where the ear pollination is not easily influenced by the climate. AJ525 represented large kernel size because its KL and KW values both ranked second, whereas A350 represented small kernel size due to the two-trait values of it belonging to the bottom 10% in the association panel. The three-trait value of AJ525 was consistently higher than that of A350 across all environments. The population structure analysis revealed that AJ525 and A350 were assigned to one subgroup.

Maize kernel filling includes three stages: a lag phase of minimal gain in dry weight (10–18 DAP), a linear phase of dry weight accumulation (18–40 DAP), and a period of diminishing dry weight accumulation approaching physiological maturity (40–70 DAP; Seebauer et al., 2010; Wang et al., 2012). The lag and linear phages are important for the formation of maize kernel size. Therefore, 20 seeds of each inbred line were collected at DAP15 and DAP39, immediately frozen in liquid nitrogen, and stored at −80°C until RNA extraction. Three biological replicates were conducted for AJ525 and A350. The RNA sequencing platform was Illumina HiSeq X Ten (BioMarker Technologies Co., Ltd., Beijing, China). Clean reads were aligned to B73_RefGen_v4^2^ using HISAT (Kim et al., 2015). The RNA sequencing data have been uploaded to the Sequence Read Archive of the National Center for Biotechnology Information (accession no. PRJNA681326).^3^

Fragments per kilobase of transcript per million fragments mapped (FPKM) was used as the gene expression level. The differential expression analysis was implemented by DESeq2 (Love et al., 2014). Genes with FPKM ≥ 1 in at least three samples were used for each pairwise comparison. The false discovery rate (FDR) < 0.05 and |log_2_fold change| ≥ 1 were set as the thresholds for significantly differential expression. Gene Ontology (GO) enrichment analysis of the differentially expressed genes (DEGs) was performed by the GOseq R package-based Wallenius noncentral hypergeometric distribution (Young et al., 2010). The KOBAS software was used to test the statistical enrichment of DEGs in KEGG pathways (Mao et al., 2005). The GATK2 software (McKenna et al., 2010) was used to perform SNP calling based on the following criteria: the quality by depth > 2 and the number of single-base mismatch within 35 bp < 3.

## Results

### Phenotype Descriptions for Kernel Size Traits

**Figure 1:**
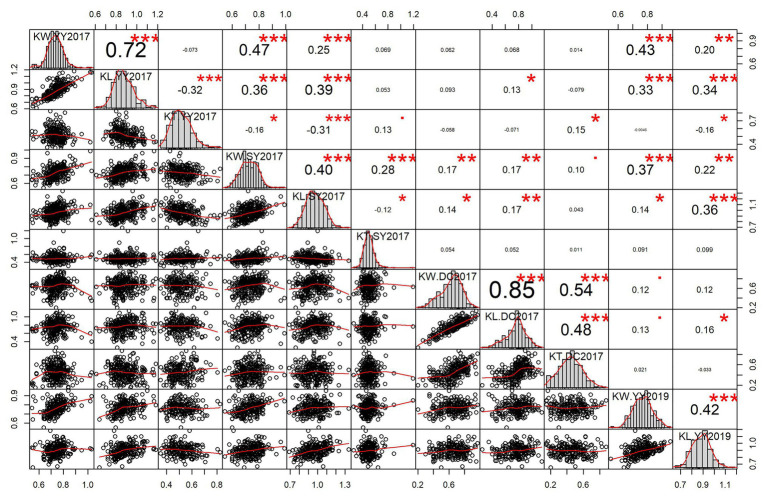
Pairwise correlations and histograms of kernel-related traits in different environments. Pearson correlation coefficient and significant level denoted by asterisk are shown in the upper triangular matrix. Significant levels at 0.05, 0.01, and 0.001 are represented by *, **, and ***, respectively. Histograms and scatter plots of kernel length (KL), kernel width (KW), and kernel thickness (KT) are shown in diagonal and lower triangular matrix, respectively. DC2017, Dancheng in 2017; SY2017, Sanya in 2017; YY2017 and YY2019, Yuanyang in 2017 and 2019. BLUE, best linear unbiased estimate.

Significant pairwise correlations were observed between the three kernel-related traits and different environments. The positive correlation between KL and KW was high in YY2017 (*r* = 0.72) and DC2017 (*r* = 0.85) and moderate in SY2017 (*r* = 0.40) and YY2019 (*r* = 0.42; Figure 1). KL was negatively correlated with KT in YY2017 (*r* = −0.32) and SY2017 (*r* = −0.12), indicating the trade-offs between them. The correlation between KL and KT was significantly positive in DC2017 (*r* = 0.48, *p* < 0.001). KW significantly and positively correlated with KT in DC2017 (*r* = 0.54, *p* < 0.001) and SY2017 (*r* = 0.28, *p* < 0.001). For KL and KW, a significantly positive correlation (*r* = 0.34–0.47, *p* < 0.001) was observed between YY2017, SY2017, and YY2019. DC2017 showed no or weak correlations with other environments for the three traits (Figure 1).

In a single environment or multi-environment, the broad-sense heritability of KL was the highest (0.65–0.97), followed by KW (0.62–0.94), and KT was the lowest (0.49–0.83; Supplementary Table S1). For the three traits, the heritability of a multi-environment was lower than that of a single environment (Supplementary Table S1). ANOVA of multi-environments showed that differences of genotype were highly significant (*p* < 0.001; Supplementary Table S2). Environment and GEI effect were also highly significant in multi-environment ANOVA (Supplementary Table S2). In order to reduce the environment effect, the BLUE value was estimated and also used as an environment for the following analysis.

### Significant Loci and Candidate Genes Identified in Association Mapping

**Figure 2:**
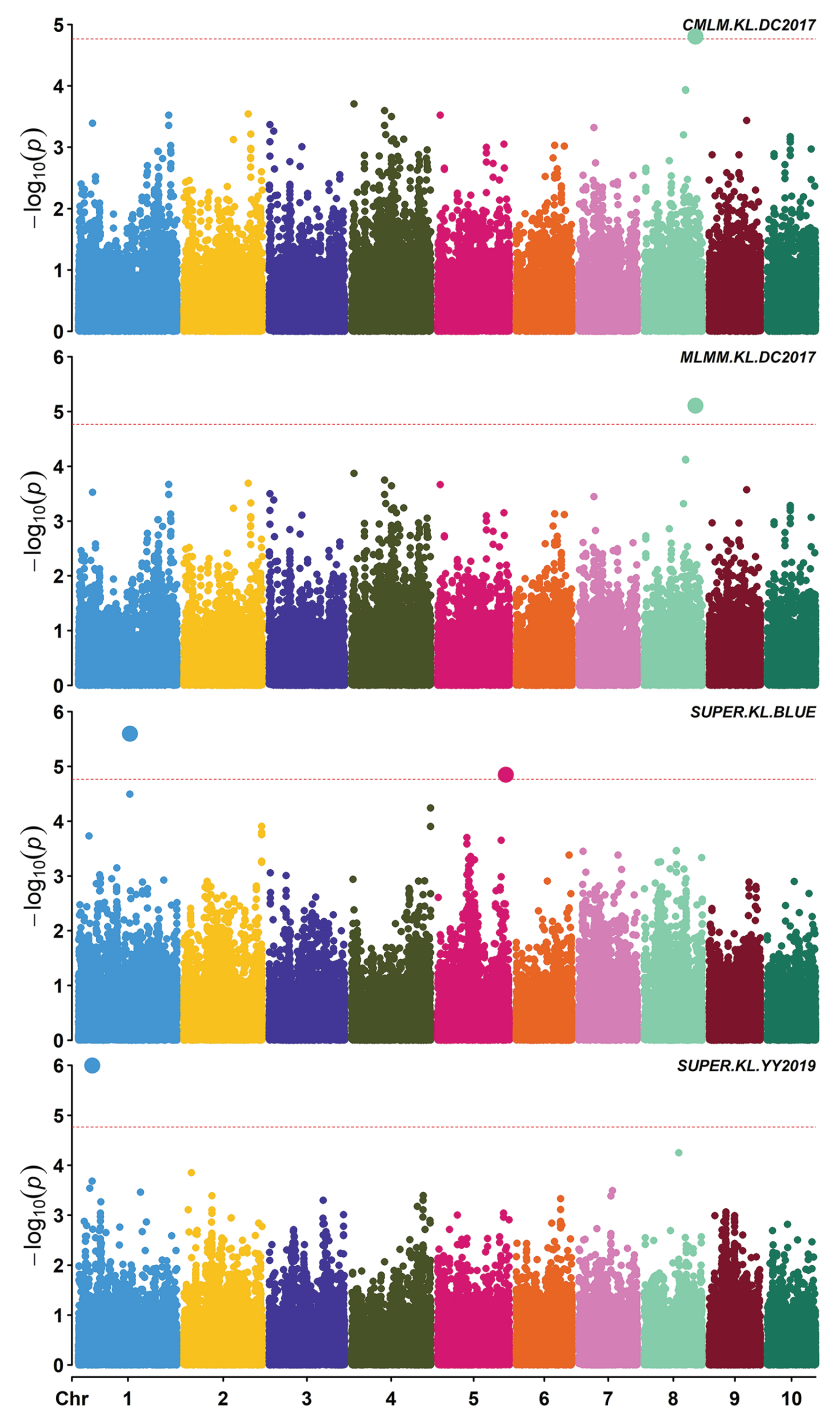
Manhattan plots of significant association analysis for KL in different environments using compressed mixed linear model (CMLM), multiple locus mixed linear model (MLMM), and settlement of MLM under progressively exclusive relationship (SUPER). The red dotted line indicates the significance threshold of *p* value 1.72E-05. DC2017, Dancheng in 2017; YY2019, Yuanyang in 2019. BLUE, best linear unbiased estimate.

**Figure 3:**
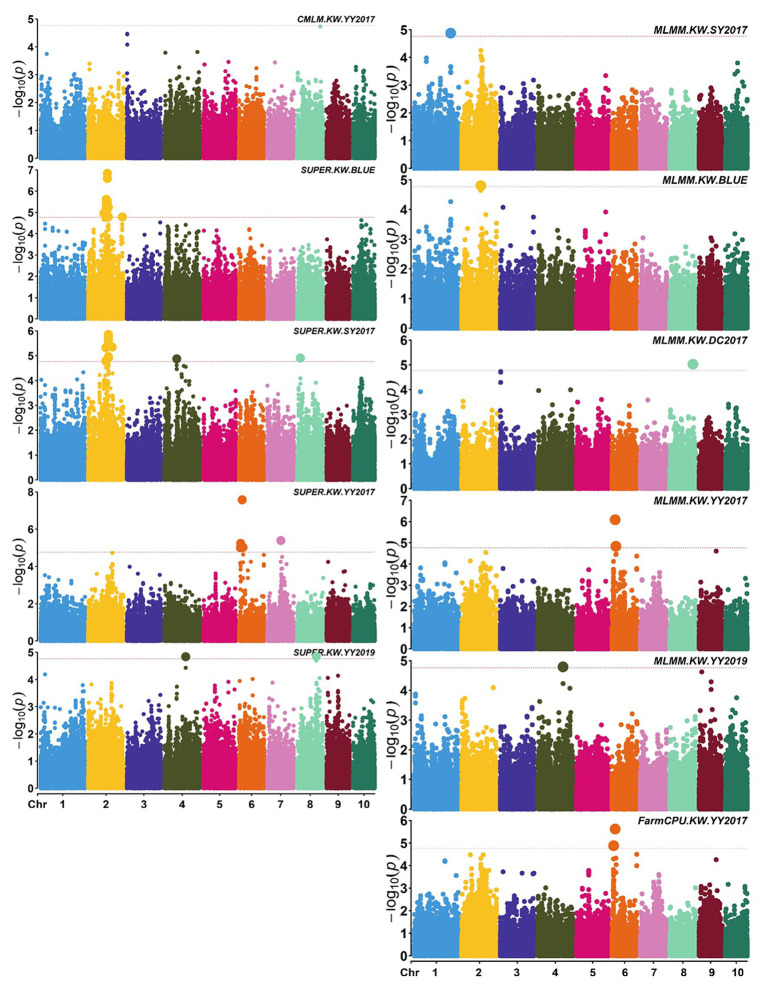
Manhattan plots of significant association analysis for KW in different environments using CMLM, SUPER, MLMM, and FarmCPU. The red dotted line indicates the significance threshold of *p* value 1.72E-05. DC2017, Dancheng in 2017; SY2017, Sanya in 2017; YY2017 and YY2019, Yuanyang in 2017 and 2019. BLUE, best linear unbiased estimate.

**Figure 4:**
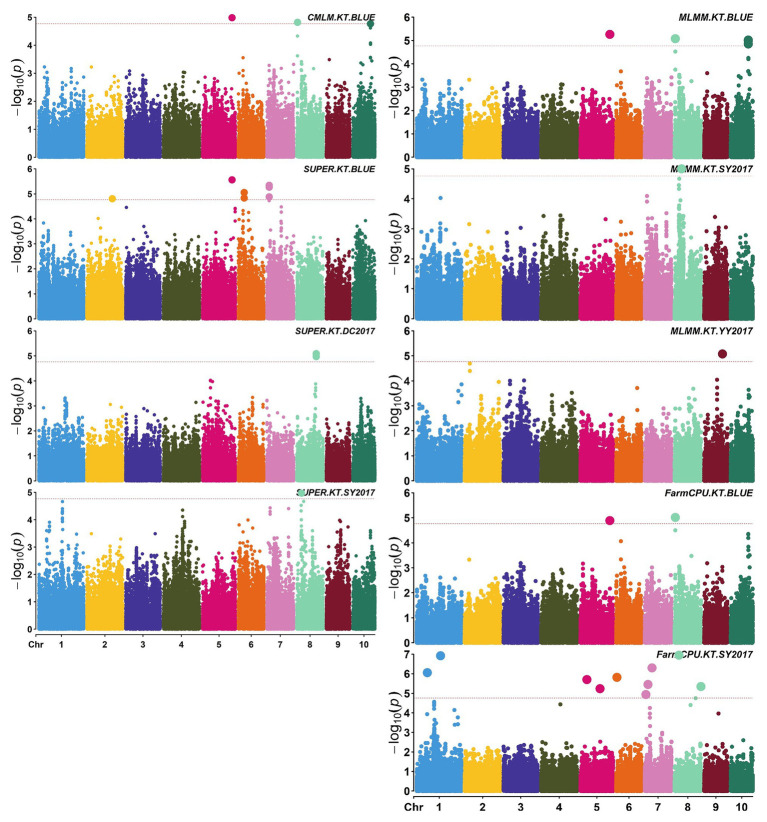
Manhattan plots of significant association analysis for KT in different environments using CMLM, SUPER, MLMM, and FarmCPU. The red dotted line indicates the significance threshold of *p* value 1.72E-05. DC2017, Dancheng in 2017; SY2017, Sanya in 2017; YY2017, Yuanyang in 2017. BLUE, best linear unbiased estimate.

**Figure 5:**
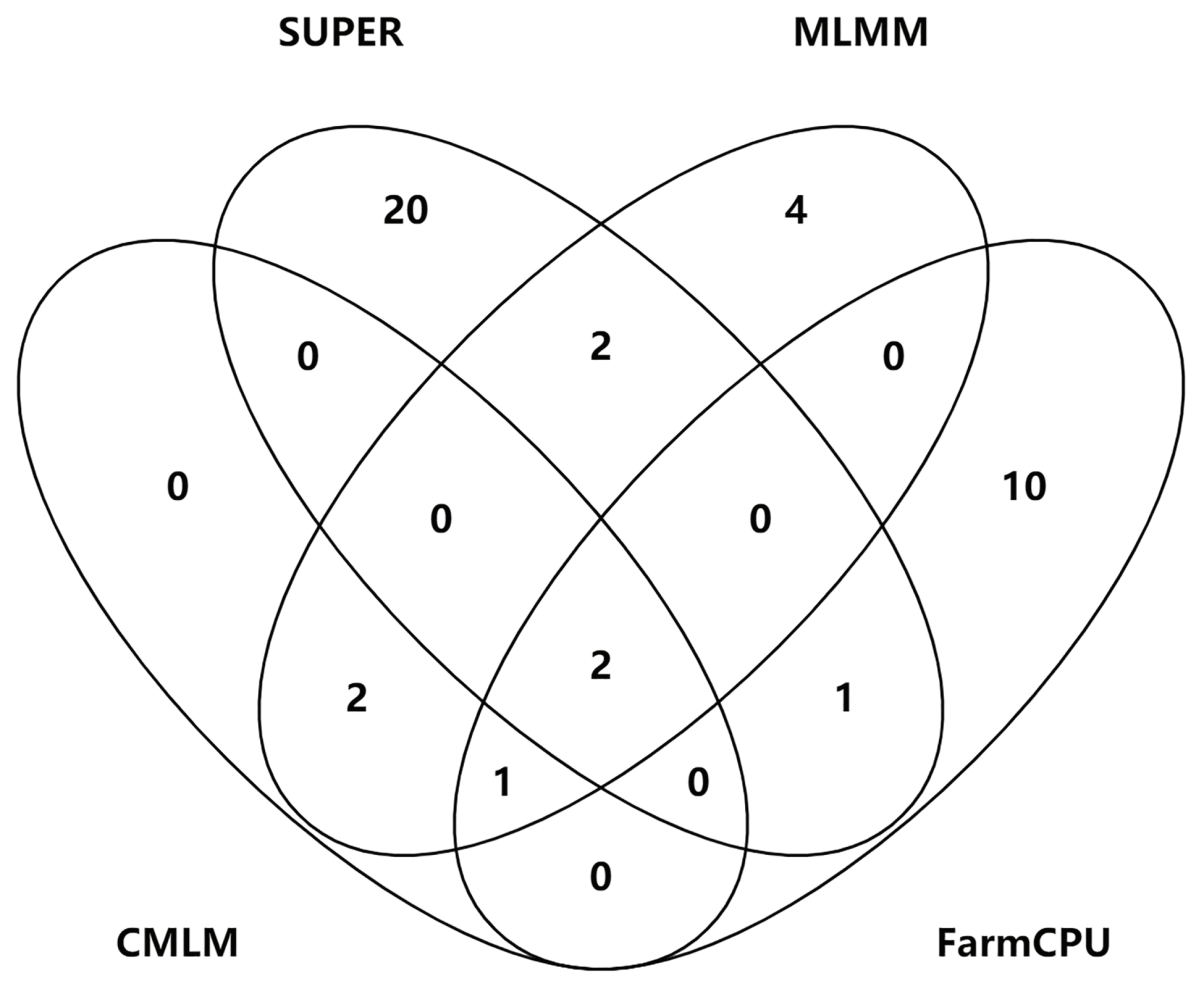
Venn plots of significant SNPs for kernel-related traits identified by four methods.

A total of 58,129 high-quality SNPs were used for summary description. The average marker density was approximately 36 kb per SNP, and the average distance between adjacent SNPs was 2.59 kb (Supplementary Figure S2A). Little variations were observed among 10 chromosomes in terms of MAF, missing rate, and heterozygous rate. The average MAF, missing rate, and heterozygous rate were 0.047, 0.051, and 0.23 across chromosomes (Supplementary Figure S2B). The number of SNPs varied from 3,932 on chromosome 10 to 7,869 on chromosome 1. Two single-locus methods (CMLM and SUPER) and two multi-locus methods (FarmCPU and MLMM) were used to balance false signals in the four planting environments and the combined environment (BLUE). A total of 42 significant SNPs (*p* < 1.72 × 10^−5^) for three traits were identified under five environments, of which five were detected by CMLM, 25 by SUPER, 14 by FarmCPU, and 11 by MLMM (Figures 2–5; Supplementary Figure S3; Supplementary Table S3). There were 3, 19, and 20 significantly associated with KL, KW, and KT, respectively, explaining 0.087–10.35% of the phenotypic variation (Supplementary Table S3).

S2_138681134 was the only one environment-stable SNP, which was co-detected in BLUE and SY2017 environment in the SUPER model (Supplementary Table S3). It could explain 8.20–8.44% of the variation for KW. Eight SNPs were method-stable which were co-identified by at least two methods, and they explained 5.87–9.59% of the phenotypic variation (Figure 5; Supplementary Table S3). S5_201017684 and S6_22359567 were co-detected by the four GWAS methods. Specifically, S5_201017684 was significantly associated with KT in BLUE (CMLM, *p* = 1.05E-05; FarmCPU, *p* = 1.29E-05; MLMM, *p* = 5.47E-06; SUPER, *p* = 2.75E-06), whereas S6_22359567 was significantly associated with KW in YY2017 (CMLM, *p* = 2.16E-06; FarmCPU, *p* = 2.39E-06; MLMM, *p* = 8.15E-07; SUPER, *p* = 2.67E-08). KT-associated SNP S8_5131540 was detected by CMLM (*p* = 1.53E-05), FarmCPU (*p* = 9.68E-06), and MLMM (*p* = 8.37E-06) in BLUE. Five SNPs were co-detected by two methods (Figure 5). Among them, S8_159308804 was co-detected by CMLM (DC2017, *p* = 1.58E-05) and MLMM (DC2017, *p* = 7.83E-06) in terms of KL. It was also significantly associated with KW (DC2017, *p* = 9.47E-06) by MLMM, indicating the SNP had a pleiotropic effect on KL and KW. S8_159308804 explained 7.18–8.58 and 8.38% of the variation of KL and KW, respectively.

We used these 42 significant SNPs to identify candidate genes for kernel-related traits, and 70 candidate genes were found (Supplementary Table S3). Four TFs—AP2-EREBP-transcription factor 10 (EREB10), EREB16, Alfin-like-transcription factor 10 (ALF10), and NAC-transcription factor 113 (NAC113)—were involved for KT, and two TFs—Aux/IAA-transcription factor 6 (IAA6) and Myb-related protein1 (MYBR1)—were involved for KW. Four E3 ubiquitin-protein ligases (RHY1A, BRE1-like 2, UPL4, and XBAT31), three ribosomal proteins (RPS8, RPS4A, and RPL7), ethylene receptor homolog40 (ERH40), proliferating cell nuclear antigen2 (PCNA2), pentatricopeptide repeat-containing protein (PPR), probable protein phosphatase 2C 33, cytochrome P450 71D7, and GDP-L-galactose phosphorylase 1 were also candidate genes for the three traits.

### Global Transcriptome Analysis of Two Inbred Lines With Contrasting Kernel Size

From the association panel, we selected two inbred lines with contrasting kernel size from field trials. In large seed AJ525, the mean value of KL, KW, and KT was 1.11, 0.85, and 0.54 cm, respectively, across different environments (Supplementary Figure S4). For A350, the mean value of KL, KW, and KT was 0.77, 0.68, and 0.50, respectively, across different environments. The difference of AJ525 and A350 was significant for the three traits in SY2017 (Supplementary Figure S4). In terms of KL, the difference between the two lines was significant in YY2017 and YY2019. KW of AJ525 was significantly higher than that of A350 in YY2019 (Supplementary Figure S4). We constructed RNA sequencing on their seeds at DAP15 and DAP39 with three replicates. The high correlation among three biological replicates indicated the reliability of our transcriptomic profiling data (Supplementary Figure S5).

To identify genes associated with kernel size, we performed pairwise comparisons between AJ525 and A350 at each development stage. The DEGs were identified with FDR < 0.05 and |log_2_fold change| ≥ 1. There were 5,156 and 8,280 DEGs between the two genotypes at DAP15 and DAP39, respectively, indicating that the major difference between the two genotypes was shown at DAP39 (Supplementary Figure S6). At DAP15, 2,630 and 2,526 genes were upregulated and downregulated, respectively, in AJ525 relative to A350. Among them, 229 TFs showed more than two-fold expression between the two genotypes, of which NAC-transcription factor 61 (NAC61), bHLH-transcription factor 38 (bHLH38), agamous-like MADS-box AGL8-like protein (AGL8L), GRAS-transcription factor 3 (GRAS3), MYBR110, and SBP-transcription factor 4 (SBP4) increased over 30-fold expression in large seed AJ525 relative to A350 (Supplementary Figure S7; Supplementary Table S4). bHLH96, E2F-DP-transcription factor 214 (E2F14), and GRAS51 showed over 16-fold expression in A350 compared with AJ525. At DAP39, 3,831 and 4,449 DEGs were upregulated and downregulated, respectively, in large seed AJ525 compared with A340 (Supplementary Figure S6). Among them, 364 TFs showed more than two-fold expression between the two genotypes, of which GRAS3, E2F14, E2F8, NAC100, NAC61, MYBR110, MYBR58, bHLH38, WRKY-transcription factor 15 (WRKY15), and AGL8L showed high differential expression in AJ525 relative to A340 (Supplementary Table S5).

Apart from these TFs, we found 86 genes encoding ribosomal proteins, 33 genes encoding PPR, and 59 genes associated with ubiquitin-conjugating enzyme, and ubiquitin-protein ligase showed over two-fold difference between AJ525 and A350 at DAP15 (Supplementary Figure S7). At DAP39, 50 genes encoding RP, 27 genes encoding PPR, and 41 genes related to ubiquitin-conjugating enzyme and ubiquitin-protein ligase were differentially expressed between the two genotypes (Supplementary Figure S7). The three family members are reported to regulate seed size in maize, rice, and *Arabidopsis* (Song et al., 2007; Xia et al., 2013; Li et al., 2014; Qi et al., 2019; Dai et al., 2020).

In the two inbred lines, DEGs between two development stages were also identified. In large seed AJ525, there were 5,452 DEGs in DAP39 stage compared with DAP15, of which sugars will eventually be exported and transporter12a showed increased expression (Supplementary Table S6). In A350, 10,316 DEGs were identified between the two stages, of which floury3, bHLH123, WRKY10, GRAS47, embryo specific protein5, and cytochrome P450 family 81 subfamily D polypeptide8 showed high differential expression (Supplementary Table S7).

Some known genes for kernel development and size including Miniature1 (*Mn1*; Kang et al., 2009), small kernel2 (*SMK2*; Yang et al., 2017), and defective kernel44 (*Dek44*; Qi et al., 2019) showed over two-fold differential expression between genotypes or development stages (Supplementary Figure S8). *Mn1* first downregulated at DAP15, and then upregulated at DAP39 in large seed AJ525 relative to A350. In addition, *Mn1* was significantly downregulated at DAP39 compared with DAP15 in both genotypes. In both genotypes, *SMK2* showed increased expression at DAP39 in relative to DAP15. At DAP39, *Dek44* showed 2.17-fold increased expression in AJ525 compared with A350.

GO analysis revealed that DNA integration, oxidation-reduction process, cell proliferation, abscisic acid/gibberellin biosynthetic process, carbohydrate metabolic process, carbohydrate transport, microtubule-based movement, and flavonol biosynthetic process were remarkable biological processes in DEGs between the genotypes and development stages (Supplementary Figure S9). KEGG analysis indicated these DEGs were mostly related to plant hormone signal transduction, starch and sucrose metabolism, glycolysis/gluconeogenesis, photosynthesis, carbon metabolism, phenylpropanoid biosynthesis, and flavonoid biosynthesis (Supplementary Figure S10).

### Candidate Genes Identified by GWAS and Transcriptome

We identified common genes detected by GWAS and transcriptome analysis. Thirty-four genes detected by GWAS were expressed (FPKM ≥ 1) in at least three samples, and 26 genes were expressed in all 12 samples. Twenty-two genes were differentially expressed between the two inbred lines or between the two development stages (Table 1; Supplementary Tables S8, S9). Three and eight genes showed significantly differential expression between the two genotypes at DAP15 and DAP39, respectively (Table 1). At DAP15, JRG21 and ureide permease 5 (UPS5) showed 4.74–6.16-fold decreased expression in AJ525 relative to A350, whereas Zm00001d020107 had presence/absence variation DEG which was only expressed in AJ525. At DAP39, ERH40, plasma membrane intrinsic protein2 (PIP2), and BRI1-KD interacting protein 130 (Zm00001d009041) showed upregulated expression in AJ525 compared with A350. Zm00001d028675 (plant-specific domain TIGR01589 family protein), Zm00001d051180 (DUF3755 family protein), UPS5, and aldehyde oxidase5 (AO5) were downregulated 2.87–10.95-fold in AJ525 compared with A350.

**Table 1:** Candidate genes identified by GWAS were significantly differentially expressed between AJ525 and A350 at DAP15 and DAP39.

DAP	Gene ID	Description	FPKM_AJ525_	FPKM_A350_	FDR value
DAP15	Zm00001d020107	Unknown	2.03	0.00	1.17E-11
	Zm00001d035462	Jasmonate-regulated gene 21 (JRG21)	2.13	8.50	5.89E-47
	Zm00001d035214	Ureide permease 5 (UPS5)	0.73	3.98	6.18E-29
DAP39	Zm00001d020107	Unknown	5.58	0.10	2.96E-18
	Zm00001d028675	Plant-specific domain TIGR01589 family protein	0.53	2.85	9.7E-13
	Zm00001d051180	DUF3755 family protein, partial	17.34	47.12	5.85E-89
	Zm00001d009041	BRI1-KD interacting protein 130	1.95	0.01	1.95E-17
	Zm00001d035214	Ureide permease 5 (UPS5)	0.45	4.96	2.56E-39
	Zm00001d018869	Aldehyde oxidase5 (AO5)	8.68	51.82	0
	Zm00001d004372	Ethylene receptor homolog40 (ERH40)	4.27	2.02	1.74E-12
	Zm00001d005421	Plasma membrane intrinsic protein2 (PIP2)	62.91	15.17	1.00E-133

Ten and 17 genes identified by GWAS were significantly differentially expressed between the two stages in the two genotypes (Supplementary Tables S8, S9). In both genotypes, MYBR1, serine/threonine-protein kinase RUNKEL, Zm00001d035222 (cell wall protein IFF6-like), and probable beta-D-xylosidase 7 (BXL7) were downregulated in DAP39 compared with DAP15, whereas E3 ubiquitin-protein ligase RHY1A and AO5 showed increased expression in DAP39 relative to DAP15. EREB16, RPS8, RPL7, JRG21, and PCNA2 also showed over two-fold differential expression between the two stages. In addition, RPL7, JRG21, serine/threonine-protein kinase RUNKEL, EREB16, and Zm00001d035222 (cell wall protein IFF6-like) were detected from the environment-table and method-stable SNPs, which may be important for regulating kernel size and development.

The allele variations detected by GWAS and transcriptome analysis were identified through 22 common genes. The positions of SNPs from 22 common genes did not match exactly in both omics levels. With a distance of 0.20–71 kb, 23 SNPs detected in the transcriptome showed consistent allele variations as five significant SNPs identified by GWAS. Of these, 20 transcriptome SNPs corresponding to three significant SNPs by GWAS showed different alleles between the two genotypes at DAP39, whereas three transcriptome SNPs from two GWAS SNPs exhibited allele variations between the two development stages in A350 (Supplementary Table S10).

## Discussion

Kernel size is a complex quantitative trait and is coordinately regulated by kernel length, width, and thickness. Elucidation of the variation of kernel size will facilitate to reveal the regulatory mechanisms of maize kernel development. GWAS and transcriptome analysis are effective methods to identify key loci and genes for kernel size in different omics levels. The combination of GWAS and transcriptome analysis is useful to improve the efficiency of gene identification.

To control false associations, two single-locus methods (CMLM and SUPER) and two multi-locus methods (MLMM and FarmCPU) were used to identify significant SNPs for kernel size. Forty-two significant SNPs for the three kernel traits were identified. Eight SNPs were co-detected by at least two methods, of which two were detected by four methods, one by three methods, and five by two single-locus and multi-locus methods. However, most of the significant SNPs were specific to each GWAS method. Similar results were also found in previous studies. Li et al. (2018) found 15 loci of 342 significant SNPs for cotton fiber quality traits were simultaneously identified in both single-locus and multi-locus models. Only two SNPs were co-detected by MLM, FarmCPU, and least absolute shrinkage and selection operator in maize starch pasting properties (Xu et al., 2018). Several studies demonstrated that multi-locus models have higher power and accuracy levels for QTL detection when compared with some single-locus models (Wang et al., 2016; Wen et al., 2017; Li et al., 2018; Xu et al., 2018; Liu et al., 2020a). Our study showed that the statistical power of SUPER was the highest, followed by FarmCPU, and CMLM was the lowest. The ways of dealing with sample size, marker size, and effects varied differently in each GWAS method, which could result in the differences of the statistical power and accuracy levels. Eight method-stable loci demonstrated that the combination of single-locus and multi-locus methods could help improve the reliability of GWAS.

Due to a significant GEI effect, only one SNP was co-detected in two environments. Although KL and KW showed a significant and positive correlation in four environments, only one SNP (S8_159308804) was detected with a pleiotropic effect on KL and KW. Except KL and KW, the pleiotropism between KW and KT or KL and KT was also found in other studies (Liu et al., 2014, 2017, 2020a). In some environments, trade-offs between KT and KL were observed (Figure 1). However, no evidence was detected in genomic level since no common SNPs were found. The PVE of 42 SNPs ranged from 0.087 to 10.35% and only one was a major effect SNP, which is consistent with previous studies that kernel size traits are mainly controlled by minor effect loci (Chen et al., 2016; Raihan et al., 2016; Liu et al., 2020a). Therefore, genomic selection rather than marker-assisted selection is useful for the application of these SNPs in maize breeding.

Twelve SNPs were located within QTL regions for kernel size in previous studies. S5_201017684 detected by four methods and S8_159308804 detected by three methods were located within QTL regions for KT, KL, and KW that were identified by Liu et al. (2017; Supplementary Table S3). S9_124772115 was a major effect SNP for KT with PVE of 10.35% and was located in qkl9, qkw9, and qhkw9 (Shi et al., 2017; Supplementary Table S3). S1_159584490 (KL) was located in a major QTL for KL (qkl1-2, PVE = 17.8%) and a KW QTL (qkw1; Shi et al., 2017). S4_176126505 for KW was identified within the intervals of qkw4 (Shi et al., 2017). S8_159308804 for KL was detected in qKL8 which had a pleiotropic effect on KW, kernel volume, and thousand kernel weight (Zhang et al., 2017). S2_106835164 (KW) and S5_134065604 (KT) were detected in two KL-associated QTL regions found by Liu et al. (2020a; Supplementary Table S3).

Three SNPs were located closely to the identified SNPs from previous studies (Supplementary Table S3). S2_234183333 for KW was detected in the BLUE environment, and at 76 kb downstream of this SNP, Li et al. (2019) found a significant SNP for KW in a NAM population. In an association panel consisting of 639 inbred lines, Li et al. (2019) found a KW SNP located at 176,210,546 bp, and in our study, a significant KW SNP at 176,126,505 bp was found on chromosome 4. In this association panel, a KL SNP at 131,861,727 bp on chromosome 2 was identified by Li et al. (2019), and at its 32 kb downstream, a KW SNP (S2_131894424) with PVE of 9.08% was found in the BLUE environment using SUPER and MLMM. We also found that 24 SNPs were located in the interval regions of meta-QTLs which were integrated in previous QTL mapping studies on kernel size traits, ear-related traits, and grain yield per plot or plant (Chen et al., 2017; Supplementary Table S3). Among them, eight SNPs for KT and KL on chromosome 2 were located in MQTL-10, and this region was only associated with kernel size traits (Chen et al., 2017). These SNPs validated by different genetic backgrounds may be important for maize kernel size and should be given more attention in genomic selection breeding.

Seventy candidate genes were identified from 42 significant SNPs, and 22 GWAS genes showed significantly differential expression between genotypes or stages (Table 1; Supplementary Tables S8, S9). In particular, five DEGs, namely EREB16, RPL7, JRG21, serine/threonine-protein kinase RUNKEL, and Zm00001d035222 (cell wall protein IFF6-like), were identified from one environment-stable and eight method-stable SNPs. EREB16 and MYBR1 showed over two-fold difference between stages. Pang et al. (2019) found that EREB170 and EREB115 were involved in kernel development in an integrated eQTL analysis. Through combined association and linkage mapping, five co-located genes annotated as MYBR1 were significantly associated with KL and KT (Liu et al., 2020a). ZmMRP-1, the first transfer cell-specific transcriptional activator, contains a MYB-related DNA binding domain and plays roles in the regulation of endosperm and transfer cell differentiation (Gómez et al., 2002).

RPL7 was a candidate gene for KW and Zm00001d047262 encoding RPS8 was identified for KT. They were significantly differentially expressed between the two development stages in A350. In addition, the corresponding SNP of RPL7 was S2_138681134 which was co-detected in two environments and considered a stable SNP for KW. Maize *Dek44* encodes mitochondrial RPL9 and regulates cell growth and kernel development *via* cyclin/cyclin-dependent kinase-mediated activities (Qi et al., 2019). *dek* mutants, a major type of maize kernel mutants, are utilized to investigate seed development. Most of the Dek genes encode PPR proteins, which are involved in seed development (Zhu et al., 2019; Dai et al., 2020). In the present study, Zm00001d007534 encoding PPR protein was associated with KW. Liu et al. (2020a) found that Zm00001d025152 encodes the PPR protein and was a candidate gene for KT identified by GWAS.

Hormone-related genes JRG21, ERH40, and BRI1 (brassinosteroid insensitive 1)-KD (kinase domain) interacting protein 130 were candidate genes for KW, and they showed significantly differential expression between the two genotypes. Ethylene receptor genes CM-ERT1 and Cm-ERS1 play a role in the early development of melon fruit (Sato-Nara et al., 1999). BR-deficient or BR-insensitive mutants resulted in small seeds, whereas overexpression of BR synthetic genes produced large seeds (Zuo and Li, 2014; Li and Li, 2015). However, the family members of JRG21, serine/threonine-protein kinase RUNKEL, and Zm00001d035222 (cell wall protein IFF6-like) have not been reported to regulate kernel size and development.

In addition, three E3 ubiquitin-protein ligase RHY1A, BRE1-like 2, and XBAT31 were candidate genes for KT, whereas E3 ubiquitin-protein ligase UPL4 was detected from a method-stable SNP (S6_27627826) for KW. Only RHY1A showed significantly differential expression between two development stages in the two inbred lines. Liu et al. (2020a) found that one gene (Zm00001d004898) encoding E3 ubiquitin-protein ligase HRD1A was significantly associated with KL. *OsGW2* encodes a RING-type E3 ubiquitin ligase and negatively regulates cell division, resulting in a decrease of grain width and weight (Song et al., 2007). *DA2*, the homology of *OsGW2*, encodes E3 ubiquitin ligase activity and regulates seed size by restricting cell proliferation in the maternal integuments of developing seeds (Xia et al., 2013). E3 ubiquitin-protein ligase RHY1A, BRE1-like 2, and XBAT31 are RING-type proteins and may have similar function in controlling seed development as *OsGW2*. The KW-associated gene Zm00001d005421, encoding plasma membrane intrinsic protein2 (PIP2), was highly expressed in AJ525 and showed significantly two-fold differential expression compared with A350. In soybean, GmPIP2-9-overexpressing plants had significantly more pod numbers and larger seed sizes than wild-type plants (Lu et al., 2018).

In summary, 42 significant SNPs for KW, KL, and KT were identified. In particular, one and eight SNPs were co-detected in two environments and by at least two methods, respectively. GWAS combined with transcriptome data revealed that RPL7, JRG21, serine/threonine-protein kinase RUNKEL, EREB16, and Zm00001d035222 (cell wall protein IFF6-like) were important candidate genes for kernel size and development.

## Data Availability Statement

The original contributions presented in the study are publicly available. This data can be found at: SRA database of NCBI (accession no. PRJNA681326).

## Author Contributions

JM constructed the phenotypic measurement and data analyses including phenotypic analysis, kinship analysis, and GWAS analysis and wrote the manuscript. JM, LW, and YC collected the seeds of 12 samples for transcriptome sequencing. HL, LW, and HW provided the 309 inbred lines. All authors contributed to the article and approved the submitted version.

### Conflict of Interest

The authors declare that the research was conducted in the absence of any commercial or financial relationships that could be construed as a potential conflict of interest.

## Footnotes

^1^http://ftp.ensemblgenomes.org/vol1/pub/plants/release-36/fasta/zea_mays/dna/Zea_mays.AGPv4.dna.toplevel.fa.gz

^2^http://plants.ensembl.org/Zea_mays/Info/Index

^3^https://www.ncbi.nlm.nih.gov/sra/PRJNA681326
