# Supplementary material for: Association Mapping and Transcriptome Analysis Reveal the Genetic Architecture of Maize Kernel Size
Source: Front Plant Sci. 2021 Mar 18;12:632788. doi: 10.3389/fpls.2021.632788 (PMC8013726; doi:10.3389/fpls.2021.632788)
Supplement: Supplementary file 1 [file Data_Sheet_1.pdf]

Supplementary Material

A

B

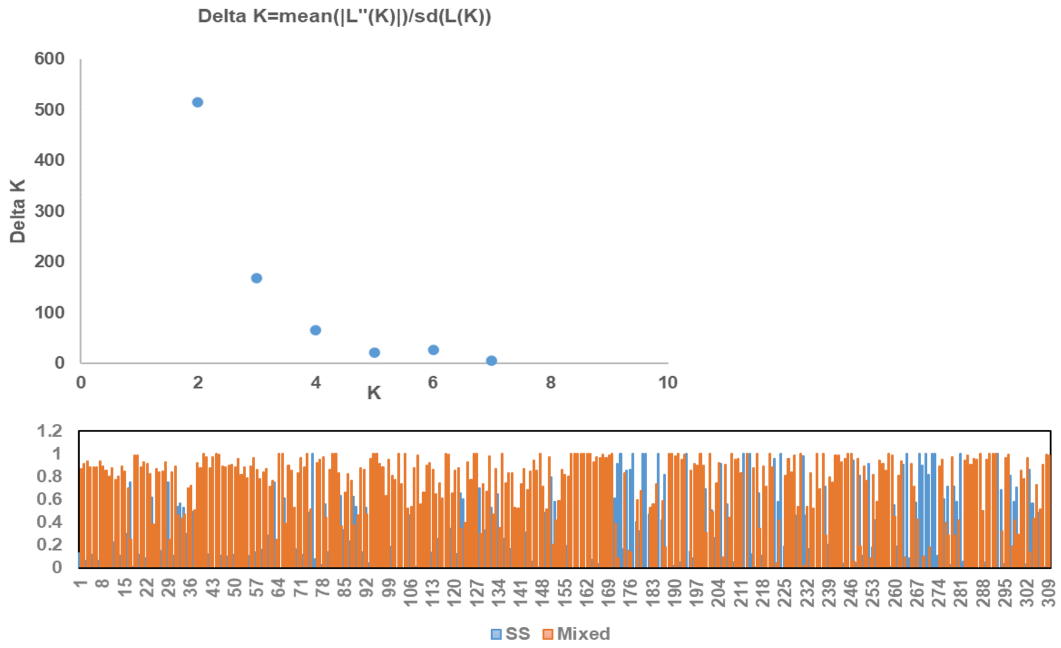

Figure S1 Population structure analysis of 309 inbred lines based on 58,129 SNPs. A: The plot of Delta K value calculated from K=1-8. The Delta K from K=1 and K=8 is not available. B: The plot of population structure from K=2. SS: Stiff Stalk; Mixed: a mixed heterotic group which is mainly comprised of non-Stiff Stalk and Reid.

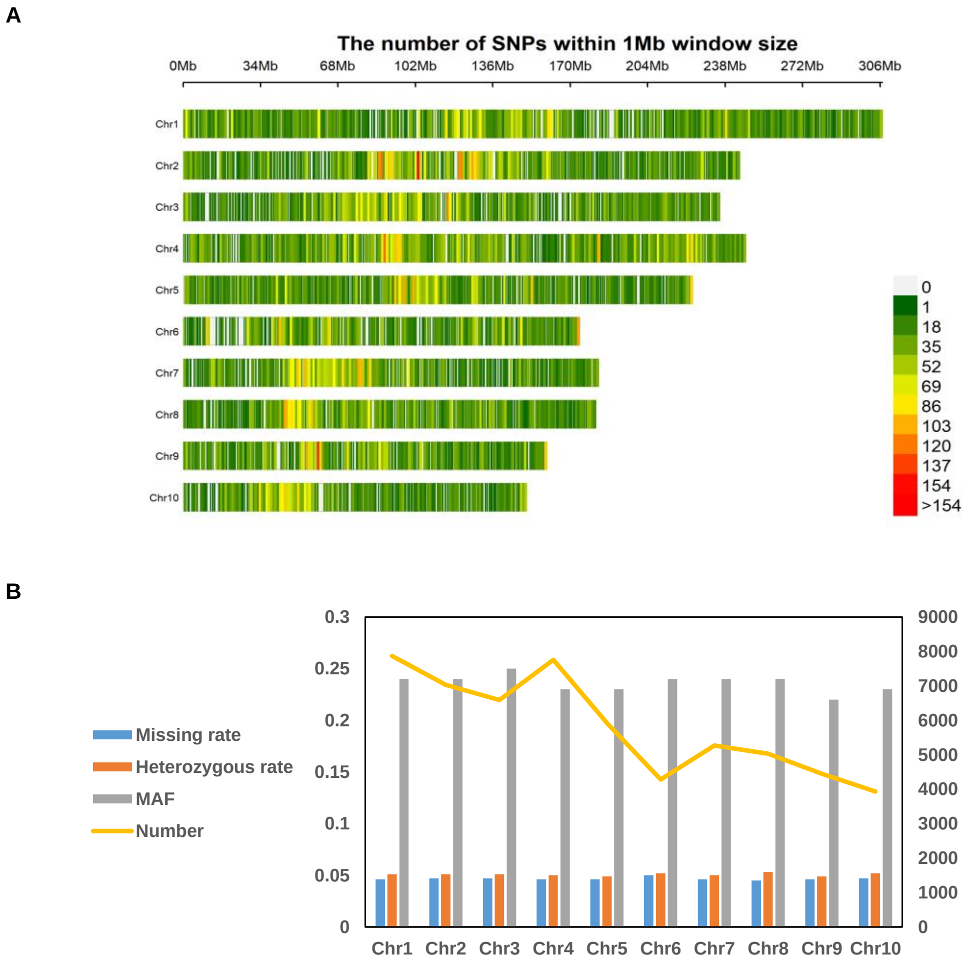

Figure S2 Summary statistics of 58,129 SNPs used for genotyping 309 inbred lines. A: SNP densities across chromosomes (Chr) B: the statistics of average missing rate, average heterozygous rate, and average minor allele frequency (MAF) of ten chromosomes, and number of SNPs on ten chromosomes.

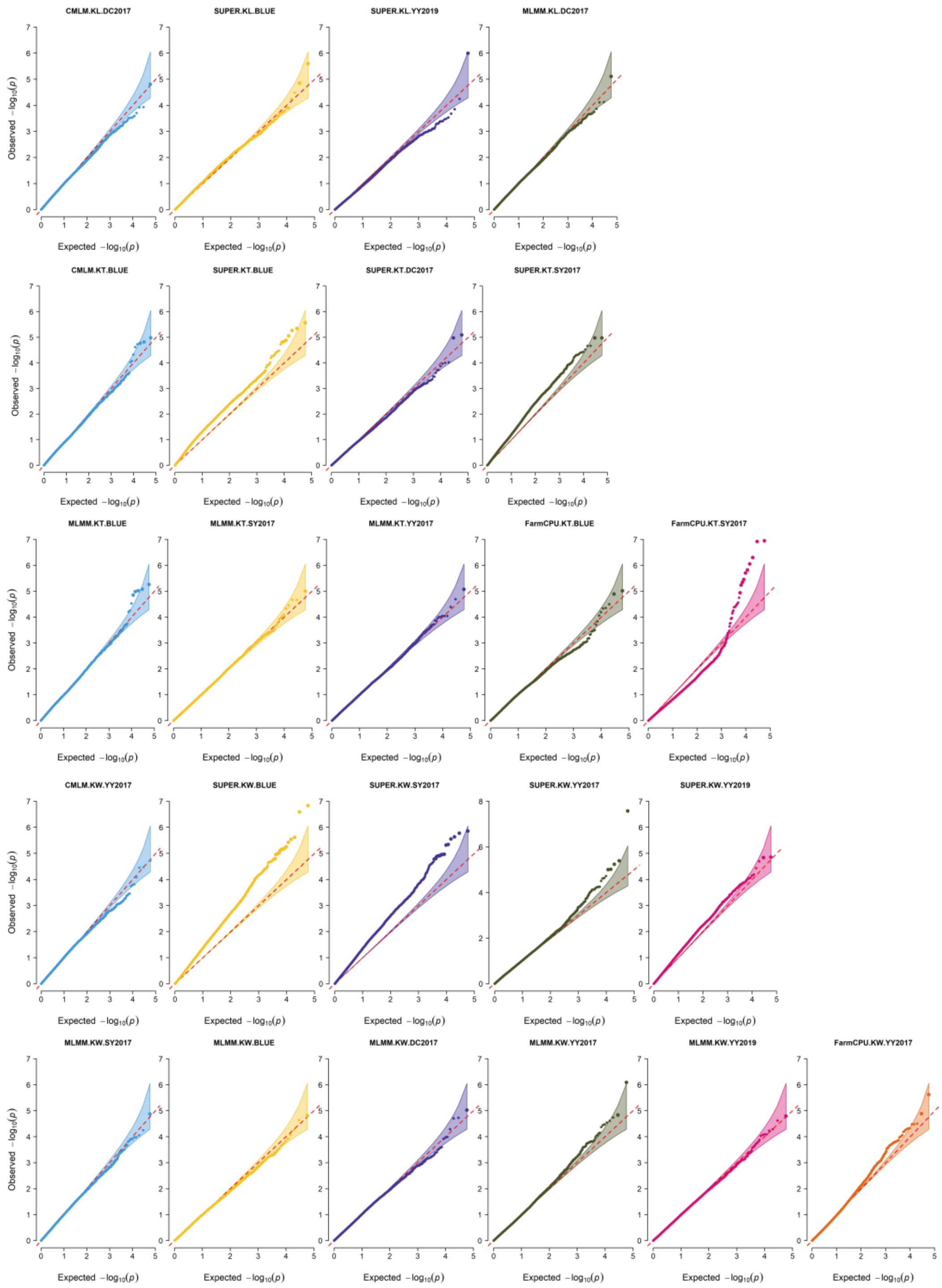

Figure S3 Quantile-Quantile plots of kernel length (KL), kernel width (KW), and kernel thickness (KT) in different environments by compressed mixed linear model (CMLM), settlement of MLM under progressively exclusive relationship (SUPER), multiple loci mixed linear model (MLMM), and fixed and random model circulating probability unification (FarmCPU). These plots are corresponding to Figure 2, Figure 3, and Figure 4. Expected null distribution of *p*-value assuming no associations, represents as solid red line. DC2017: Dancheng in 2017; SY2017: Sanya in 2017; YY2017 and YY2019: Yuanyang in 2017 and 2019; BLUE: Best linear unbiased estimate.

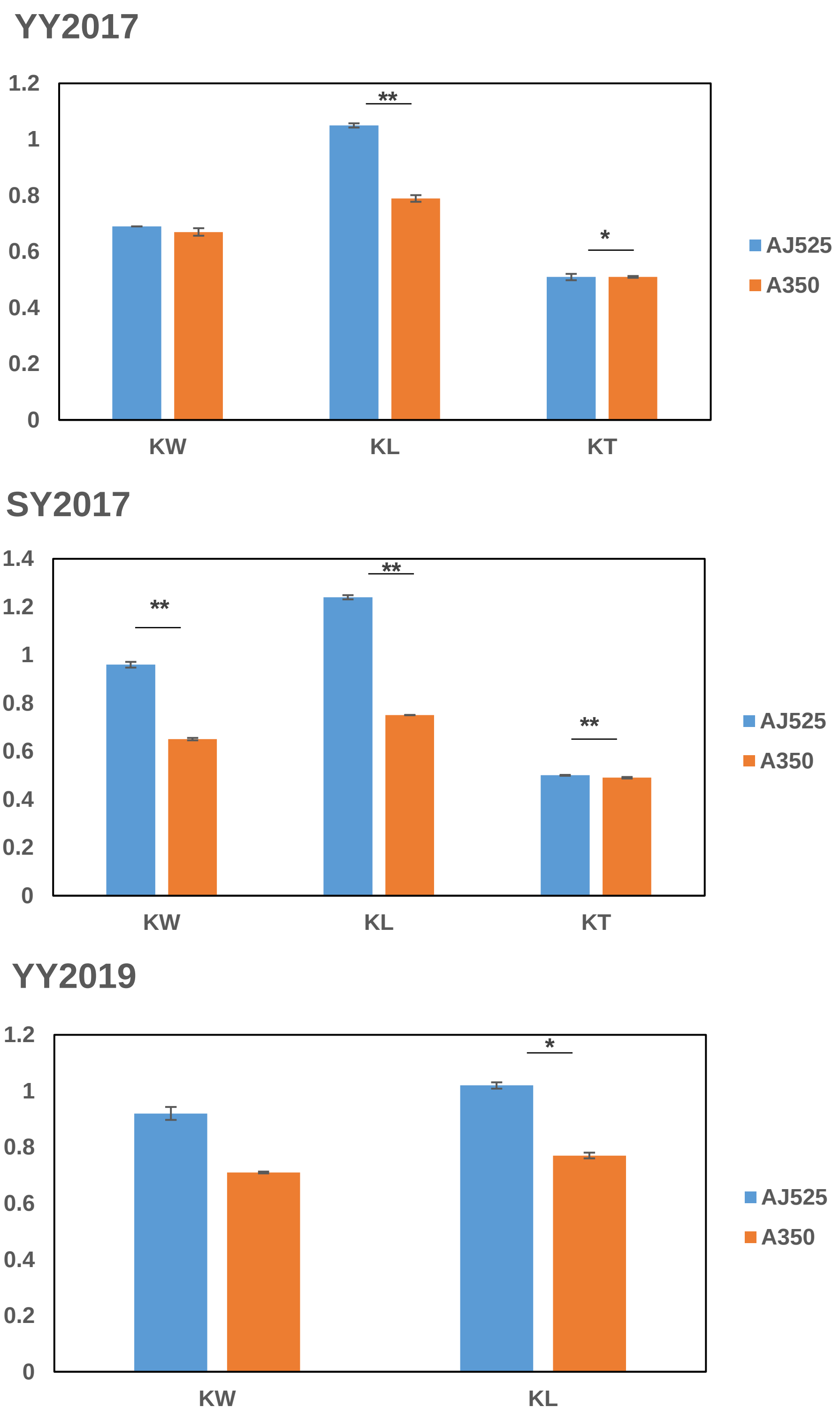

Figure S4 Performance of KL, KW, and KT of AJ525 and A350 in YY2017, SY2017, and YY2019. * and ** mean the differences are significant at the 0.05 and 0.01 levels, respectively.

DAP15	AJ525-1	AJ525-2	AJ525-3
AJ525-1	1		
AJ525-2	0.983723	1	
AJ525-3	0.989321	0.996255	1
DAP15	A350-1	A350-2	A350-3
A350-1	1		
A350-2	0.98824	1	
A350-3	0.991214	0.991272	1
DAP39	AJ525-1	AJ525-2	AJ525-3
AJ525-1	1		
AJ525-2	0.985551	1	
AJ525-3	0.99478	0.993393	1
DAP39	A350-1	A350-2	A350-3
A350-1	1		
A350-2	0.993942	1	
A350-3	0.994622	0.993304	1

Figure S5 Pearson correlations between three biological replicates for AJ515 and A350 at DAP15 and DAP39.

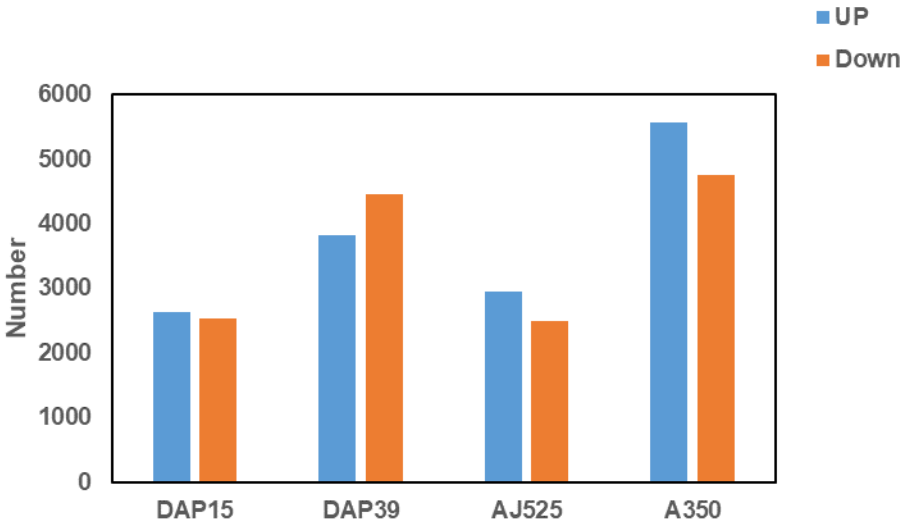

Figure S6 Number of upregulated and downregulated significant differentially expressed genes between the two genotypes and two stages. DAP15 and DAP39 represent the differentially expressed genes in AJ525 compared with A350 at DAP15 and DAP39, respectively. AJ525 and A350 represent the differentially expressed genes in DAP39 compared with DAP15 in AJ525 and A350, respectively.

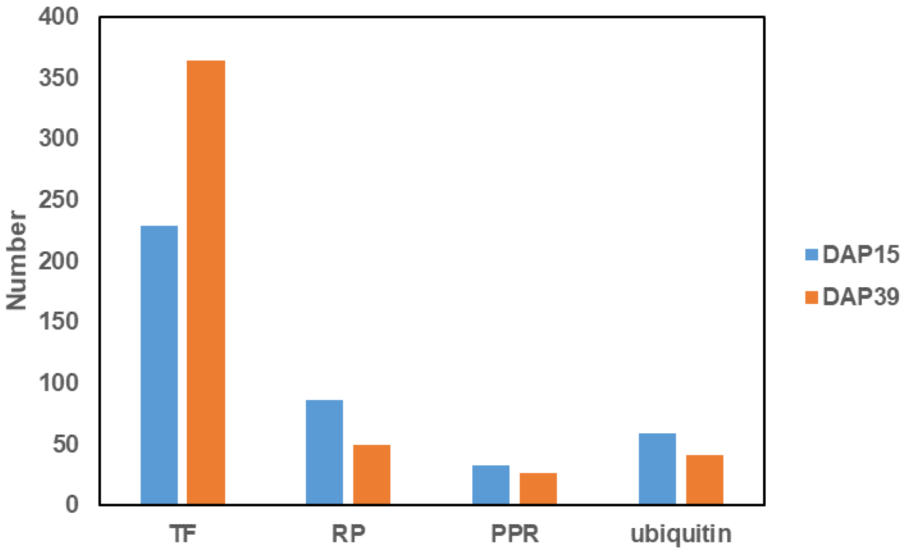

Figure S7 Number of transcription factors (TF), ribosomal proteins (RPs), pentatricopeptide repeat-containing protein (PPR), ubiquitin-related genes including ubiquitin-conjugating enzyme and ubiquitin-protein ligase showing significantly differentially expressed between the two genotypes at DAP15 and DAP39.

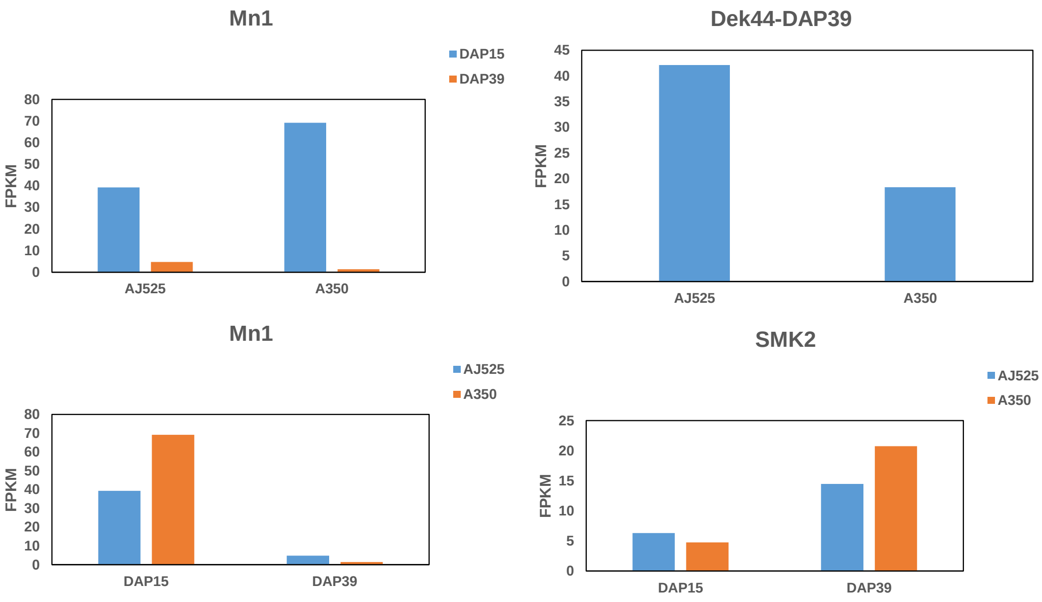

Figure S8 FPKM values of *Mn1*, *Dek44*, and *SMK2* between the two genotypes at DAP15 and DAP39 and between the two stages in AJ525 and A350.

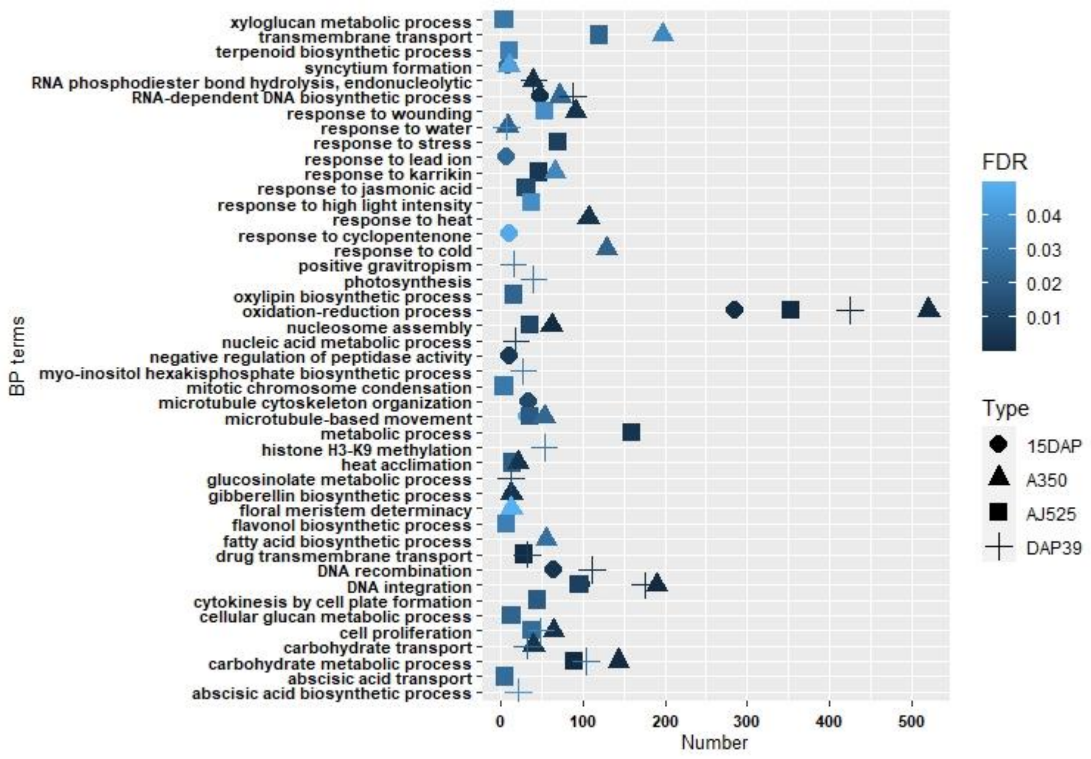

Figure S9 Significant biological processes for differentially expressed genes between the two genotypes and two stages. DAP15 and DAP39 represent BP terms for differentially expressed genes between AJ525 and A350 at DAP15 and DAP39, respectively. AJ525 and A350 represent BP terms for differentially expressed genes between DAP15 and DAP39 in AJ525 and A350, respectively

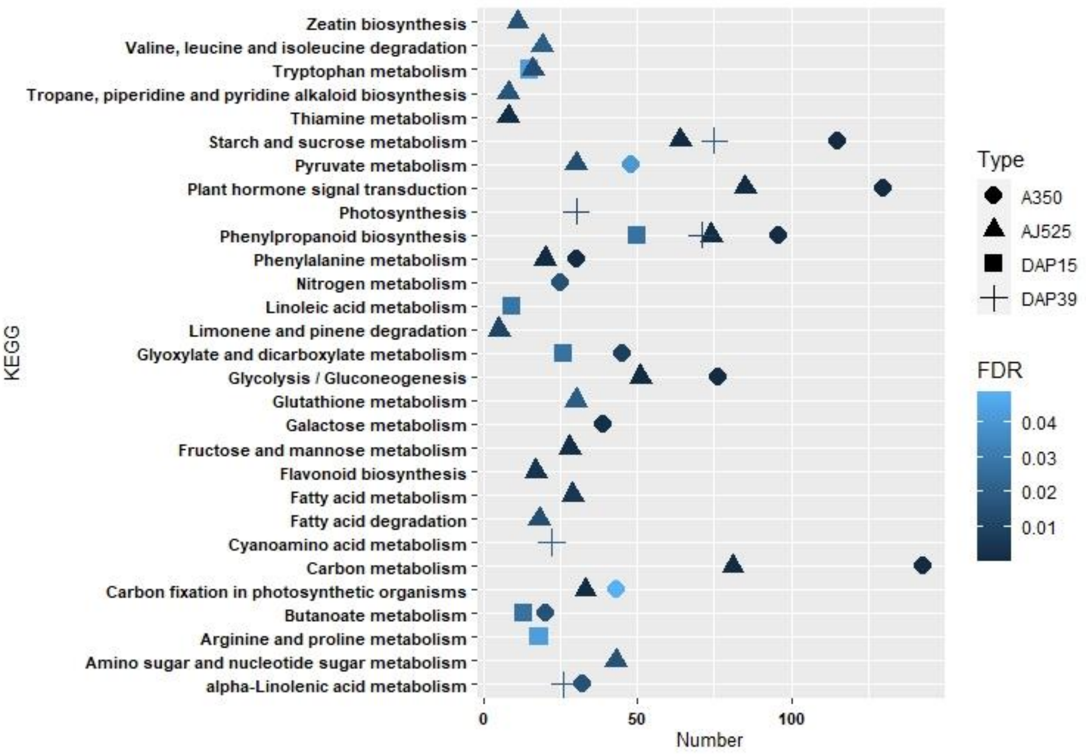

Figure S10 Significant KEGG pathways for differentially expressed genes between the two genotypes and two stages. DAP15 and DAP39 represent KEGG pathways for differentially expressed genes between AJ525 and A350 at DAP15 and DAP39, respectively. AJ525 and A350 represent KEGG pathways for differentially expressed genes between DAP15 and DAP39 in AJ525 and A350, respectively.

Table S1 Heritability of KL, KW, and KT in single environment and multi-environment

Environment	KW	KL	KT
YY2017	0.83	0.85	0.70
SY2017	0.94	0.97	0.55
DC2017	0.84	0.88	0.83
YY2019	0.78	0.82	-
Multi-environment	0.62	0.65	0.49

Table S2 Multi-environment variance of analysis for KL, KW, and KT

Traits	Sources	Degree of freedom	Sum of square	Mean of square	F value	P value
KW	Block/Environment	8	0.024	0.003	1.019	0.42
	Genotype	308	9.39	0.031	10.48	0
	Environment	3	4.17	1.39	477.31	0
	Genotype and environment interaction	766	12.94	0.017	5.81	0
	Error	1763	5.13	0.0029		
KL	Block/Environment	8	0.043	0.0053	1.69	0.097
	Genotype	308	15.30	0.050	15.72	0
	Environment	3	13.47	4.49	1421.73	0
	Genotype and environment interaction	766	18.95	0.025	7.83	0
	Error	1762	5.57	0.0032		
KT	Block/Environment	5	0.088	0.018	2.84	0.015
	Genotype	308	8.77	0.029	4.61	0
	Environment	2	1.31	0.66	106.37	8.05E-06
	Genotype and environment interaction	483	11.26	0.023	3.77	0
	Error	965	5.96	0.0062		

Table S3 Significant SNPs and candidate genes for KL, KW, and KT in different environments using CMLM, SUPER, MLMM, and FarmCPU

SNP	Methods	Chromosome	Position (bp)	P value	MAF	PVE(%)	Trait-environment combinations	Candidate genes	Reference
S1_159584490	SUPER	1	159584490	2.52E-06	0.09	6.78	KL.BLUE	Zm00001d030784,Zm00001d030785	Shi et al. 2017; Liu et al. 2017
S1_162831127	FarmCPU	1	162831127	1.20E-07	0.25	5.73	KT.SY2017	Zm00001d030843(RHY1A), Zm00001d030845(CYP71D7)	
S1_252128523	MLMM	1	252128523	1.33E-05	0.40	6.73	KW.SY2017	Zm00001d033148	Liu et al. 2017; Chen et al. 2017
S1_42659928	SUPER	1	42659928	1.01E-06	0.25	6.31	KL.YY2019	Zm00001d028673,Zm00001d028675	Li et al. 2019
S1_72903936	FarmCPU	1	72903936	8.79E-07	0.39	5.13	KT.SY2017	Zm00001d029490(BRE1-like2)	
S2_106835164	SUPER	2	106835164	1.11E-05	0.30	3.86	KW.BLUE	Zm00001d004371(Senescence-specific cysteine protease SAG12), Zm00001d004372(ERH40)	Liu et al. 2020a; Chen et al. 2017
S2_123443410	SUPER	2	123443410	3.79E-06	0.49	5.51	KW.BLUE	Zm00001d004626(BXL7), Zm00001d004630(phosphatidylethanolamine-binding protein17)	Chen et al. 2017
S2_123494286	SUPER	2	123494286	2.42E-06	0.50	5.77	KW.BLUE	Zm00001d004626(BXL7), Zm00001d004630(phosphatidylethanolamine-binding protein17)	Chen et al. 2017
S2_123608845	SUPER	2	123608845	1.01E-05	0.47	4.83	KW.BLUE	Zm00001d004626(BXL7), Zm00001d004630(phosphatidylethanolamine-binding protein17)	Chen et al. 2017
S2_131894424	SUPER	2	131894424	1.47E-07	0.30	9.08	KW.BLUE	Zm00001d004692, Zm00001d004697(IAA6)	Chen et al. 2017
	MLMM			1.56E-05					
S2_138681134	SUPER	2	138681134	5.58E-06	0.45	8.20	KW.BLUE	Zm00001d004779(RPL7), Zm00001d004782(transducin/WD40 repeat-like superfamily protein)	Chen et al. 2017
				1.39E-06		8.44	KW.SY2017		
S2_138704456	SUPER	2	138704456	1.69E-06	0.44	7.61	KW.SY2017	Zm00001d004779(RPL7), Zm00001d004782(transducin/WD40 repeat-like superfamily protein)	Chen et al. 2017
S2_138704614	SUPER	2	138704614	1.61E-05	0.44	7.40	KW.BLUE	Zm00001d004779(RPL7), Zm00001d004782(transducin/WD40 repeat-like superfamily protein)	Chen et al. 2017

S2_173456581	SUPER	2	173456581	1.56E-05	0.15	1.65	KT.BLUE	Zm00001d005421(PIP2), Zm00001d005422(FAD/NAD(P)-binding oxidoreductase family protein)	Chen et al. 2017
S2_234183333	SUPER	2	234183333	1.63E-05	0.46	3.58	KW.BLUE	Zm00001d007534(PPR), Zm00001d007535	Li et al. 2019
S4_147473140	SUPER	4	147473140	1.45E-05	0.12	2.24	KW.YY2019	Zm00001d051180,Zm00001d051182	
S4_176126505	MLMM	4	176126505	1.61E-05	0.47	3.60	KW.YY2019	Zm00001d051995(PCNA2), Zm00001d051998(AP-1 complex subunit gamma-1)	Shi et al. 2017; Li et al. 2019
S5_134065604	FarmCPU	5	134065604	5.77E-06	0.27	0.087	KT.SY2017	Zm00001d015955(Topoisomerase II-associated protein PAT1), Zm00001d015956	Liu et al. 2020a
S5_201017684	SUPER	5	201017684	2.75E-06	0.09	6.89	KT.BLUE	Zm00001d017591(EREB10), Zm00001d017592	Liu et al. 2017
	MLMM			5.47E-06					
	FarmCPU			1.29E-05					
	CMLM			1.05E-05		6.44			
S5_212356885	SUPER			1.42E-05	0.24	1.87	KL.BLUE	Zm00001d018025	
S5_44593901	FarmCPU	5	44593901	1.99E-06	0.10	0.38	KT.SY2017	Zm00001d014400, Zm00001d014405(NAC113)	Li et al. 2019; Liu et al. 2017; Chen et al. 2017
S6_10216527	SUPER	6	10216527	9.95E-06	0.06	7.42	KW.YY2017	Zm00001d035214(UPS5), Zm00001d035215(RPS4A)	
S6_11527125	SUPER	6	11527125	5.83E-06	0.05	8.26	KW.YY2017	Zm00001d035217,Zm00001d035222	
	FarmCPU			1.31E-05					
S6_22359567	SUPER	6	22359567	2.67E-08	0.07	9.59	KW.YY2017	Zm00001d035337(GDP-L-galactose phosphorylase 1), Zm00001d035342	
	MLMM			8.15E-07					
	FarmCPU			2.39E-06					
	CMLM			2.16E-06		8.22			
S6_27627826	SUPER	6	27627826	9.58E-06	0.07	7.52	KW.YY2017	Zm00001d035461(UPL4), Zm00001d035462(JRG21)	
	MLMM			1.46E-05			KW.YY2017		
S6_39314400	SUPER	6	39314400	8.80E-06	0.07	0.76	KT.BLUE	Zm00001d035656, Zm00001d035657(XBAT31)	Chen et al. 2017
S6_4737681	FarmCPU	6	4737681	1.52E-06	0.21	2.52	KT.SY2017	Zm00001d035089	Liu et al. 2017; Chen et al. 2017

S7_15073411	SUPER	7	15073411	4.57E-06	0.22	2.87	KT.BLUE	Zm00001d019067	Liu et al. 2017; Chen et al. 2017
S7_21757447	FarmCPU	7	21757447	3.52E-06	0.17	1.75	KT.SY2017	Zm00001d019200	Liu et al. 2017; Chen et al. 2017
S7_49292383	FarmCPU	7	49292383	5.01E-07	0.10	4.71	KT.SY2017	Zm00001d019668,Zm00001d019669(cinnamoyl CoA reductase2)	Liu et al. 2017
S7_7860016	FarmCPU	7	7860016	1.15E-05	0.08	1.02	KT.SY2017	Zm00001d018869(AO5)	
S7_92340985	SUPER	7	92340985	4.10E-06	0.25	4.89	KW.YY2017	Zm00001d020105, Zm00001d020107	Liu et al. 2017
S8_132321301	SUPER	8	132321301	1.40E-05	0.15	2.40	KW.YY2019	Zm00001d010889(MYBR1), Zm00001d010892	Liu et al. 2017; Chen et al. 2017
S8_132715151	SUPER	8	132715151	8.13E-06	0.16	7.56	KT.DC2017	Zm00001d010903,Zm00001d010904(PP2C33)	Chen et al. 2017
S8_159308804	MLMM	8	159308804	9.47E-06	0.11	8.38	KW.DC2017	Zm00001d011706(probable mitochondrial-processing peptidase subunit beta)	Zhang et al. 2017; Liu et al. 2017; Chen et al. 2017
	MLMM			7.83E-06		8.58	KL.DC2017		
	CMLM			1.58E-05		7.18	KL.DC2017		
S8_180051817	FarmCPU	8	180051817	4.49E-06	0.11	2.37	KT.SY2017	Zm00001d012760(Golgin candidate)	
S8_30560636	FarmCPU	8	30560636	1.12E-07	0.08	7.70	KT.SY2017	Zm00001d009022,Zm00001d009023	Chen et al. 2017
S8_32329435	SUPER	8	32329435	1.07E-05	0.15	8.97	KT.SY2017	Zm00001d009041,Zm00001d009046	Chen et al. 2017
S8_47439834	MLMM	8	47439834	1.00E-05	0.07	8.74	KT.SY2017	Zm00001d009250,Zm00001d009253	Chen et al. 2017
S8_5131540	MLMM	8	5131540	8.37E-06	0.35	7.51	KT.BLUE	Zm00001d008321(serine/threonine-protein kinase RUNKEL)	Chen et al. 2017
	FarmCPU			9.68E-06					
	CMLM			1.53E-05		6.20			
S9_124772115	MLMM	9	124772115	8.44E-06	0.08	10.35	KT.YY2017	Zm00001d047262(RPS8)	Shi et al. 2017; Liu et al. 2017; Chen et al. 2017
S10_120760809	MLMM	10	120760809	9.56E-06	0.25	5.87	KT.BLUE	Zm00001d025504(ALF10), Zm00001d025506	Chen et al. 2017
	CMLM			1.72E-05		6.11			

Table S4 Representative differentially expressed transcription factors between AJ525 and A350 at DAP15 stage

Gene ID	Description	AJ525	A350	FDR	log2FC
Zm00001d019793	SBP4	7.32	0.00	1.06E-24	5.79
Zm00001d044311	MYBR110	1.81	0.00	3.5E-18	5.14
Zm00001d030744	GRAS3	3.45	0.00	2.7E-31	6.33
Zm00001d047742	MADS28	1.26	0.00	3.31E-14	4.65
Zm00001d015747	MYBRA	7.58	0.45	5.61E-56	3.35
Zm00001d048786	bHLH38	3.23	0.00	2.34E-19	5.26
Zm00001d027546	bHLH35	1.28	0.07	1.94E-14	3.38
Zm00001d027395	NAC78	3.04	0.19	1.27E-55	3.47
Zm00001d024543	NAC61	8.18	0.00	1.22E-61	8.13
Zm00001d045227	AGL8L	4.86	0.00	8.58E-18	5.09
Zm00001d003553	GRAS51	0.07	1.89	5.71E-29	-4.43
Zm00001d006739	LBD14	0.86	5.74	2.64E-31	-2.89
Zm00001d044272	bHLH94	0.53	2.96	1.99E-31	-2.68
Zm00001d023444	bHLH30	0.42	4.55	6.08E-18	-3.38
Zm00001d034888	WRKY10	0.24	1.92	2.68E-17	-3.00
Zm00001d026147	r1(bHLH)	0.08	1.95	0.000192	-2.62
Zm00001d052648	MADS52	3.07	28.62	2.2E-111	-3.45
Zm00001d038664	E2F14	0.06	1.25	5.4E-19	-4.08
Zm00001d004095	bHLH44	1.31	6.68	2.26E-43	-2.56
Zm00001d007382	bHLH96	0.74	22.71	2E-255	-5.14

Table S5 Representative differentially expressed transcription factors between AJ525 and A350 at DAP39 stage

Gene ID	Description	AJ525	A350	log2FC	FDR
Zm00001d041886	NAC100	8.19	0.00	6.74	1.04E-19
Zm00001d044311	MYBR110	2.74	0.00	6.93	6.64E-17
Zm00001d030744	GRAS3	6.69	0.00	8.40	5.44E-28
Zm00001d048786	bHLH38	4.79	0.00	6.89	3.1E-16
Zm00001d051480	MYBR58	1.67	0.00	6.96	4.1E-17
Zm00001d046518	MYB82	1.35	0.00	6.55	1.26E-14
Zm00001d023615	WARK15	2.04	0.00	6.83	2.64E-16
Zm00001d007248	GRAS64	1.45	0.00	6.56	1.04E-14
Zm00001d024543	NAC61	3.24	0.01	6.75	1.45E-35
Zm00001d045227	AGL8L	10.63	0.00	7.34	1.3E-19
Zm00001d005622	WRKY38	0.18	3.35	-4.00	7.03E-20
Zm00001d037221	TCP10	0.09	8.09	-5.72	7.82E-18
Zm00001d038863	bHLH87	1.87	43.54	-4.59	9.9E-278
Zm00001d012725	TCP29	0.00	1.42	-6.94	6.89E-17
Zm00001d037054	WRKY13	0.04	1.77	-4.59	2.72E-11
Zm00001d038664	E2F14	0.00	3.96	-8.13	1.18E-25
Zm00001d003451	EIL7	2.78	46.87	-4.07	1.09E-41
Zm00001d033815	MYB77	0.00	1.45	-5.72	2.26E-10
Zm00001d016907	E2F8	0.00	21.40	-9.95	3.45E-44
Zm00001d048588	GRAS	0.08	1.59	-4.15	3.88E-21

Table S6 Representative differentially expressed genes between DAP15 and DAP39 in AJ525

Gene ID	Description	DAP15	DAP39	log2FC
Zm00001d037894	RAB17 protein	5.54	4200.90	9.42
Zm00001d044967	aspartic proteinase nepenthesin-1-like	0.80	1173.94	9.99
Zm00001d052449	agmatine coumaroyltransferase-1-like	0.00	19.05	9.34
Zm00001d012663		0.04	23.86	8.41
Zm00001d048629		0.04	551.72	12.21
Zm00001d030316		14.18	0.00	-9.41
Zm00001d030314	proline-rich protein precursor	28.08	0.02	-8.72
Zm00001d019704		1.21	455.42	8.37
Zm00001d007411	NADH-ubiquinone reductase complex 1 MLRQ subunit	1.38	458.58	8.21
Zm00001d044022	seed maturation protein PM41	1.47	785.15	8.74
Zm00001d027832	embryonic protein DC-8	0.98	926.12	9.69
Zm00001d018954	Rhicadhesin receptor	66.66	0.23	-8.09
Zm00001d031228	E3 ubiquitin-protein ligase Topors-like	0.00	217.79	11.15
Zm00001d004687	O-methyltransferase ZRP4	14.32	0.00	-8.62
Zm00001d029135	sugars will eventually be exported transporter12a	0.00	8.92	8.38

Table S7 Representative differentially expressed genes between DAP15 and DAP39 in A350

Gene ID	Description	DAP15	DAP39	log2FC	FDR
Zm00001d009292	floury3	253.01	0.28	-9.96	1.1E-248
Zm00001d037528	bHLH123	2.91	0.00	-8.37	1.32E-12
Zm00001d004897	Basic-leucine zipper (bZIP) transcription factor family protein	0.01	62.05	10.77	1.77E-47
Zm00001d034888	WRKY-transcription factor 10	1.92	0.00	-8.20	5.02E-12
Zm00001d034107	cytochrome P450 family 81 subfamily D polypeptide 8	0.00	6.89	9.20	4.01E-16
Zm00001d023617	laccase-25-like precursor	0.01	51.88	11.46	2.49E-55
Zm00001d022630	knotted1 induced 1 precursor	94.18	0.00	-12.60	3.08E-36
Zm00001d025018	alpha-expansin 1 precursor	18.90	0.00	-10.67	1.2E-23
Zm00001d041498	GRAS47	0.00	3.33	8.05	1.57E-11
Zm00001d029681	lysM domain containing protein	0.12	277.51	10.42	6.49E-77
Zm00001d016152	sodium/calcium exchanger family protein / calcium-binding EF hand family protein	0.03	67.01	10.37	7.8E-109
Zm00001d037985	embryo specific protein 5	3.15	1479.41	8.52	2.4E-233
Zm00001d053237	Cation/H(+) antiporter 1	0.00	6.03	10.14	9.52E-21
Zm00001d031228	PREDICTED: E3 ubiquitin-protein ligase Topors-like	0.00	644.87	13.86	5.54E-48
Zm00001d004687	O-methyltransferase ZRP4	13.46	0.00	-10.14	1.01E-20

Table S8 Candidate genes identified by GWAS showing significantly differentially expressed between the two stages in AJ525.

Gene ID	Description	DAP15	DAP39	FDR
Zm00001d020107	unknown	2.03	5.58	5.65E-06
Zm00001d035222	cell wall protein IFF6-like	21.58	6.47	8.14E-22
Zm00001d015956	NAD(P)-binding Rossmann-fold superfamily protein	13.68	46.65	6.68E-71
Zm00001d010889	Myb related protein1 (MYBR1)	3.42	1.036	9.26E-10
Zm00001d017592	AP2-EREBP-transcription factor 16 (EREB16)	2.02	0.68	0.00049
Zm00001d009041	BRI1-KD interacting protein 130	1.01	1.95	0.00021
Zm00001d008321	Serine/threonine-protein kinase RUNKEL	3.23	1.10	2.65E-19
Zm00001d030843	Probable E3 ubiquitin-protein ligase RHY1A	2.75	6.95	1.18E-09
Zm00001d004626	probable beta-D-xylosidase 7	1.21	0.078	1.77E-20
Zm00001d018869	aldehyde oxidase5	3.16	8.68	6.39E-44

Table S9 Candidate genes identified by GWAS showing significantly differentially expressed between the two stages in A350.

Gene ID	Description	DAP15	DAP39	FDR value
Zm00001d047262	Putative ribosomal protein S8 family protein (RPS8)	15.57	7.73	8.90E-19
Zm00001d035462	jasmonate-regulated gene 21 (JRG21)	8.50	3.38	1.036E-25
Zm00001d028675	LOC100284567 isoform X1	0.12	2.85	1.05E-17
Zm00001d035222	cell wall protein IFF6-like	26.66	6.068	2.11E-47
Zm00001d051180	DUF3755 family protein, partial	12.79	47.12	2.76E-111
Zm00001d051995	proliferating cell nuclear antigen2 (PCNA2)	22.051	10.17	3.99E-39
Zm00001d010889	Myb related protein1 (MYBR1)	5.50	0.33	7.00E-36
Zm00001d008321	Serine/threonine-protein kinase RUNKEL	2.77	0.51	1.50E-31
Zm00001d030843	Probable E3 ubiquitin-protein ligase RHY1A	1.59	9.13	2.06E-27
Zm00001d004626	Probable beta-D-xylosidase 7	1.41	0.015	1.23E-20
Zm00001d051998	AP-1 complex subunit gamma-1	5.66	2.75	5.31E-22
Zm00001d018869	aldehyde oxidase5 (AO5)	4.75	51.82	0
Zm00001d004372	ethylene receptor homolog40 (ERH40)	3.42	2.020	1.75E-12
Zm00001d035089	pleckstrin homology (PH) domain-containing protein	4.55	12.06	5.15E-30
Zm00001d004779	ribosomal protein L7 (RPL7)	8.61	21.85	2.89E-24
Zm00001d005421	plasma membrane intrinsic protein2 (PIP2)	36.00	15.17	5.26E-69
Zm00001d018025	protein tyrosine kinase family protein	9.86	5.94	2.50E-15

Table S10 SNP variations detected by GWAS and transcriptome analysis

Significant SNPs by GWAS	SNPs in transcriptome	Chromosomes	Candidate genes	Information of differential expression	Alleles	AJ525-DAP15†	A350-DAP15	AJ525-DAP39	A350-DAP39
S2_106835164	106905716	2	Zm00001d004372(ERH40)	Differentially expressed between two genotypes at DAP39	C/T	C	T	C	T
S2_106835164	106906580	2	Zm00001d004372(ERH40)	Differentially expressed between two genotypes at DAP39	C/T	C	T	C	T
S4_147473140	147443484	4	Zm00001d051180	Differentially expressed between two genotypes at DAP39	C/A	A	C	A	C
S4_147473140	147444374	4	Zm00001d051180	Differentially expressed between two genotypes at DAP39	C/A	A	N	A	C
S4_147473140	147446318	4	Zm00001d051180	Differentially expressed between two genotypes at DAP39	C/A	C	A	C	A
S6_4737681	4690828	6	Zm00001d035089	Differentially expressed between two stages in A350	A/G	N	A	N	G
S6_4737681	4719877	6	Zm00001d035089	Differentially expressed between two stages in A350	A/G	N	A	G	G
S7_7860016	7850260	7	Zm00001d018869(AO5)	Differentially expressed between two genotypes at DAP39	C/T	T	C	T	C
S7_7860016	7850681	7	Zm00001d018869(AO5)	Differentially expressed between two genotypes at DAP39	C/T	T	C	T	C
S7_7860016	7853203	7	Zm00001d018869(AO5)	Differentially expressed between two genotypes at DAP39	C/T	C	T	C	T
S7_7860016	7853349	7	Zm00001d018869(AO5)	Differentially expressed between two genotypes at DAP39	C/T	T	N	T	C
S7_7860016	7853419	7	Zm00001d018869(AO5)	Differentially expressed between two genotypes at DAP39	C/T	C	T	C	T
S7_7860016	7854024	7	Zm00001d018869(AO5)	Differentially expressed between two genotypes at DAP39	C/T	N	T	C	T
S7_7860016	7854064	7	Zm00001d018869(AO5)	Differentially expressed between two genotypes at DAP39	C/T	C	T	C	T
S7_7860016	7855682	7	Zm00001d018869(AO5)	Differentially expressed between two genotypes at DAP39	C/T	T	C	T	C
S7_7860016	7856103	7	Zm00001d018869(AO5)	Differentially expressed between two genotypes at DAP39	C/T	N	T	C	T
S7_7860016	7856490	7	Zm00001d018869(AO5)	Differentially expressed between two genotypes at DAP39	C/T	T	C	T	C
S7_7860016	7857387	7	Zm00001d018869(AO5)	Differentially expressed between two genotypes at DAP39	C/T	T	C	T	C
S7_7860016	7859585	7	Zm00001d018869(AO5)	Differentially expressed between two genotypes at DAP39	C/T	N	C	T	C
S7_7860016	7859818	7	Zm00001d018869(AO5)	Differentially expressed between two genotypes at DAP39	C/T	N	C	T	C
S7_7860016	7861149	7	Zm00001d018869(AO5)	Differentially expressed between two genotypes at DAP39	C/T	N	C	T	C
S7_7860016	7861495	7	Zm00001d018869(AO5)	Differentially expressed between two genotypes at DAP39	C/T	N	T	C	T
S9_124772115	124769870	9	Zm00001d047262(RPS8)	Differentially expressed between two stages in A350	C/T	N	T	T	C

†: N represents missing. The consistent allele type in at least two biological replicates is used.
